# Prioritization decision-making of care in nursing homes: A qualitative study

**DOI:** 10.1177/09697330241230513

**Published:** 2024-02-06

**Authors:** Pauliina Hackman, Arja Häggman-Laitila, Marja Hult

**Affiliations:** 1 University of Eastern Finland; 2 South-Eastern University of Applied Sciences

**Keywords:** Aged care, decision-making, nursing home, prioritization, unfinished nursing care

## Abstract

**Background:**

Prioritization decision-making arises when nurses encounter intricate situations that demand ethically challenging judgments about care. This phenomenon has rarely been studied in nursing homes. Prioritization decision-making may lead to instances where individuals in social and healthcare may not receive all services they need. Making prioritization decisions and awareness of their consequences can increase nurses’ workload.

**Aim:**

To describe prioritization decision-making regarding unfinished nursing care in nursing homes.

**Research design:**

A qualitative descriptive study conducted through individual theme interviews. Participants were recruited through social media. The data was analyzed using inductive content analysis.

**Participants and research context:**

Nurses (*n* = 23) working in nursing homes for the elderly people in Finland. Data were collected between June 2022 and February 2023.

**Ethical considerations:**

Finnish legislation does not mandate an ethical review or research permits, as the participants took part as private individuals. [ask authors to make reference here to informed consent process and anonymity]

**Findings:**

Nurses stated that the need for prioritization decision-making arises from challenges associated with nurses’ engagement with person-centered care, the culture of the work community, the burden due to workload and challenges associated with the leadership. Prioritization decision-making was based on the interests of residents, striving for an efficient workflow and nurse’s personal interests. Nurses did not receive support for decision-making regarding unfinished care, and protocols for prioritization had not been established in their work communities. Prioritization decision-making and unfinished care were concealed and left unspoken.

**Conclusion:**

Nursing leaders should address this hidden phenomenon, making it visible through discussions with nurses and by involving them in the development of protocols. The findings can be utilized for developing new approaches to support nurses and reduce their workload and for enhancing the quality and person-centeredness of nursing care in nursing homes.

## Introduction

Nurses frequently confront the complex challenge of reconciling their workload with limited resources while striving to meet the needs of every individual in their care,^1^ requiring them to prioritize nursing care.^2^ Prioritization decision-making in nursing can be described using the term implicit rationing, in which nurses, relying on clinical decision-making, decide to withhold or not carry out necessary nursing activities for several reasons.^3^ This process can be defined as unfinished nursing care, originating from a problem (such as a lack of resources) and progressing through decision-making to an outcome in which individuals in social and healthcare may not receive all the services they need, leaving the care process unfinished.^2^ The antecedents for prioritization decision-making have been identified as a high workload, inadequate staffing, unexpected situations, and conflicting demands.^4^

The prioritization decision-making process, the core of unfinished nursing care, has been studied limitedly in hospital settings,^5^ but very sparingly in nursing homes.^4^ Over a decade ago, an analysis of descriptions obtained from Norwegian nurses and physicians in nursing homes indicated that the severity of the acute illness and the age of the resident were the antecedents of prioritization decision-making.^6^ Two recent studies from Australia have diversified these results,^7,8^ indicating that during their prioritization decision-making, nurses assess the urgency of situations, anticipation of consequences, residents’ care needs and quality of life, and balancing between safety and affording residents their independence.^8^ Prioritization decision-making is also guided by a humanistic and holistic approach to resident care^7^ and nurses’ perceived role responsibilities, teamwork, and peer support.^8^

According to a previous literature review,^4^ unfinished nursing care occurs in nursing homes on a weekly basis or even during every shift, emphasizing the crucial role of prioritization decision-making in nursing. The prevalence has been reported to vary between 29% and 75%, depending on the measurement method.^4^ This indicates that residents in nursing homes do not receive all the service and care they need, which leads to poor resident outcomes, such as residents’ urinary tract infections.^9^ The most significant deficits occur in activities related to mobility and social care,^4^ as nurses primarily prioritize medical treatment and acute monitoring.^6^ Unfinished nursing care also has consequences for nurse outcomes, as it has been observed to increase nurses’ burnout and job dissatisfaction.^10^ Other identified consequences for nurses include conflicts between their personal and professional values, which challenge nurses’ ethical and moral value systems.^5^ Decision-making creates feelings of guilt for nurses if they make decisions without support or guidelines.^5^ The support given to nurses in their prioritization decision-making has been ignored in previous studies.

This study focuses on prioritization decision-making in nursing homes. Supplementing and enhancing the existing knowledge about nurses’ prioritization decision-making in nursing homes is essential for the quality and safety of person-centered care. This information can be utilized to develop protocols for decision-making support, improve nurses’ and leaders’ capabilities in prioritization decision-making and develop new approaches to reduce nurses’ workload.

## Aim of the study

The aim of the study was to describe prioritization decision-making regarding unfinished nursing care in nursing homes.

The research questions were:

1. What kind of reasons do nurses describe as causing the need for prioritization decision-making?
2. How do nurses describe their prioritization decision-making?
3. How is nurses’ prioritization decision-making regarding unfinished nursing care supported in nursing homes?

## Methods

### Study design

A qualitative descriptive study via individual theme interviews^11^ and inductive content analysis.^12^

### Participants and research context

The participants were nurses working in nursing homes providing 24-hour care for the elderly people. The inclusion criteria were that the participants had to be able to communicate in the Finnish language and agreed to participate in the study by providing their informed consent. Participants who were not working in a nursing home or did not have nursing education were excluded.

All participants were recruited via social media. The posts included information about the voluntarity of the study participation and a link to the appointment system for choosing a suitable time for the interviews. Interviews were booked using an email address, and no other identifying information was collected. In total, 23 nurses were recruited.

### Data collection

Due to the sensitive nature of the topic, individual theme interviews were conducted.^11^ They allow participants to describe their experiences of sparsely studied topics and, thus, enable the production of new knowledge.^11^ The interview guide used was based on previous knowledge^11^ and consisted of three themes based on the research questions. The interviews were conducted in Finnish.

The interviews began with a reading of the research information and informed consent. It continued with questions about participant’s background (age, gender, education, professional experience and the province of their workplace). The interview included opening questions based on the themes. For example, the opening question for the prioritization theme was “How do you prioritize care and other tasks?” At the end of the interview, the participant was asked to relate any additional comments and describe their experience of the interview. The comprehensibility of the themes was tested through two pilot interviews that were not included in the data.

The interviews were conducted via Teams between June 2022 and February 2023. They lasted between 25 and 85 minutes (mean: 57 minutes) and were recorded with the participants’ permissions.

### Data analysis

The data were transcribed verbatim (286 pages, Times New Roman font 12, spacing 1.0) and the manifest content was analyzed through inductive content analysis, as the existing knowledge about the phenomenon was insufficient.^12^ The transcripts were read several times, during which units of analysis, such as words or phrases of original expressions that addressed the research questions, were selected.^12^ They were abridged, condensed, and grouped into subcategories based on the similarities and differences in their content. The subcategories were compared, and their differences and similarities were identified. Subcategories with similar content were grouped into the same upper category, which was named accordingly. The contents of the upper categories were compared, and main categories were established based on the similarities and differences between upper categories.^12^ During each phase, these categories were compared with the original data.

### Ethical considerations

The ethical principles of research with human participants of Finnish National Board on Research Integrity were adhered to.^13^ According to Finnish Legislation and the Finnish National Board on Research Integrity, this study did not require an ethical committee review or ethical assessment. Research permits were not required, as the participants took part in the research as private individuals. Further, the administrators of the social media groups had granted permission to publish the invitation to the study.

The participants were sent the interview themes, a privacy statement and an informed consent form in advance. All participants consented to participating in the study, and this was recorded at the beginning of the interview. Participation was voluntary and conducted anonymously. Participants were informed that they could withdraw from the study at any time without reprisal.

## Findings

In total, 23 female nurses participated on this study, of whom eight were registered nurses and 15 were practical nurses. Their age varied between 27 and 66 years (mean: 44.6 years), while their professional experience ranged from 1 to 37 years (mean: 13.3 years). The participants were from nine different provinces in Finland.

### Reasons causing the need for prioritization decision-making

Four main categories represented the reasons causing the need for prioritization decision-making in different dimensions. They were challenges associated with nurses’ engagement with person-centered care as nurse-related reasons, the culture of the work community as work community-related reasons, the burden due to workload as working-conditions-related reasons and challenges associated with leadership as leadership-related reasons (Table 1).

**Table 1.** Reasons causing the need for prioritization decision-making provided by the participating nurses (*n* = 23).

Subcategory	Upper category	Main category
Nurses’ inability to be reciprocally present with the residents Lack of nurses’ professional skills in terms of observing and reacting to residents’ well-being Nurses without permission to administer medications Deficiencies in language skills Nurses’ inadequate technology skills	Inadequacy in terms of the nurses’ competence	Challenges associated with nurses’ engagement with person-centered care
Only basic care is considered as the responsibilities of the nurses’ Nurse-oriented way to work The residents are regarded as an objects of nursing care Challenges related to prioritization of nursing care	Nurses’ incomplete understanding of the entirety of nursing care	
Staying in nurses’ own comfort zone Nurses’ negative attitude towards new things	Nurses’ insufficient capacity to change and develop	
The experience of indifference of nurses The experience of the nurses’ lack of initiative The experience of the nurses’ lack of interest	Nurses’ apathy	
Disrespect of colleagues Experience of colleagues being unsuitable for providing nursing care	Lack of collegiality	The culture of the work community
An atmosphere of fear in the work community Bad work atmosphere	Lack of psychological safety	
High threshold for asking for and giving help to colleagues Lack of collaboration	Idealization of surviving alone	
Idealization of working fast Halfway-done culture Appeals to hurry	Idealization of maximum efficiency	
Withdrawal Responsibility is assigned to certain nurses	Passive culture	
Preferring current procedures Strict way of working	The culture does not allow development	
Psychosocial burden Physical burden	Experience of the psychological and physical burden of work	The burden due to workload
Challenges in recovery during and after the working day Fatigue Irregular working hours	Challenges in recovery	
Unequal workload Strict schedule	Work scheduling challenges	
Dishes Laundry Cleaning Food preparation and serving Functions related to various orders	Non-nursing tasks carried out by nurses	
The amount of responsibility of permanent nurses increases when working with substitutes under short-term employment Limited tasks of short-term substitutes	Delegation of responsibility and tasks for permanent nurses	
Residents refuse nursing care Increased needs of residents Nurses’ resources are dedicated to residents who demand care and to those whose care is fast to perform	Complexity of residents’ needs	
Passive leader The leader is absent from the daily operations of the unit The leader’s understanding of nursing care is poor	Challenges in leaders’ actions and skills	Challenges associated with the leadership
Lack of planning unit operations Unclear job descriptions The need to perform several tasks simultaneously	Challenges related coordinating work across different tasks and roles	
Insufficient documentation of nursing care Failures in communication	Challenges associated with information flow	
New employees lack clarity regarding their duties Limited resources for nurses to constantly induct new employees	Insufficient induction of new employees	
Absences are not covered by substitutes Frequently changing short-term substitutes	Challenges in the availability and retention of nurses	
Small number of nurses relative to the amount and demands of the work Human resources are not sufficient for unexpected situations	Allocation of limited human resources	
Lack of registered nurses Lack of staff that can perform non-nursing tasks Employees without professional education	Challenges in employee structure	

*Challenges associated with nurses’ engagement with person-centered care* included a concern about inadequacy of the nurses’ ability to be present with the residents. Participants had noticed nurses’ inadequate skills to observing and responding to residents’ well-being. They emphasized that working with nurses who did not have permission to administer medications increased their workload. In addition, the participants highlighted deficiencies in nurse’s language and IT skills (i.e., using electronic record systems). They identified nurses’ incomplete understanding of the entirety of nursing care, which emerged as considering only basic care as the responsibilities of a nurse. The working approach was described nurse-oriented, and the residents were regarded as objects of nursing care.

P3: “How can we generate interest in the resident as an induvial, such that they are not perceived just as the object of nursing care but a human being with their own history and personality?”

According to the participants, some nurses prioritized non-nursing tasks over nursing care. Nurses had insufficient capacities to change and develop, which manifested as a preference to stay within their comfort zones and have a negative attitude towards change. The participants defined nurse’s apathy as indifference towards residents and a lack of initiative and interest in their work.

*The culture of the work community* in prioritization decision-making was reflected through a lack of collegiality and disrespecting of colleagues. Not all colleagues were regarded as being suitable as nurses. A lack of psychological safety was characterized as an atmosphere of fear, where no one dared to oppose it. It was also described as a bad work atmosphere with conflicts and blaming of colleagues. The idealization of surviving alone meant a high threshold for asking for and offering help as well as a lack of collaboration among colleagues.

P14: “It’s kind of like, from the very beginning, we are taught this culture of self-reliance—that you have to manage alone because rarely does anyone have time to help you.”

The idealization of maximum efficiency emerged as working fast, halfway-done culture and appeals to hurry. The participants stated that hurrying was used as an excuse for nursing care done half-heartedly.

P6: “In my opinion, that sense of hurry is partly learned. It’s also very easy to always use the excuse that I’m so busy, so I can’t. But there’s plenty of time to sit in the office. Unfortunately, that’s seen quite a lot.”

Passive culture within the work community emerged nurses withdrawing through congregating together, along with an uneven distribution of responsibilities. The participants expressed that the culture does not allow development and prefers current procedures. They described the strict way of working as a contrived and rigid culture into which new employees and students are inducted.

They described *the burden due to workload* as psychological and physical. Making significant decisions alone and staying highly alert for residents were reported to be psychologically exhausting, while assisting those with greater needs was considered physically challenging. The participants identified challenges related to recovering from their work, which were caused by the absence of breaks, long recovery times, fatigue, and irregular working hours, such as working in three shifts.

P9: “Shift work, of course, drains energy from all of us.”

According to the participants, work scheduling challenges, including unequal workloads and strict schedules, were also connected to the burden due to workload. There were peak hours during their shifts, and the schedule was based on residents’ mealtimes. They highlighted that nurses also carried out non-nursing tasks, such as those related to dishes, laundry, cleaning, food preparation, serving and functions related to various order (e.g., food orders).

P13: “Nurses then do the dishes and clean the dining halls and kitchens. Those tasks are done in between the resident’s nursing care, depending on who has time. That time could be spent better with the residents.”

Working with short-term substitutes affected the delegation of the responsibilities and tasks of permanent nurses and increased their workload. Substitutes lack familiarity with residents and adherence to unit protocols, and they carry out limited tasks focusing on basic care. The complexity of residents’ needs manifested as the refusal of care and an increased need for the same, which stem from factors such as multiple illnesses and poor health, increasing the nurses’ workload. The participants reported that nurses’ resources were dedicated to residents who demand care and whose care is fast to perform.

P21: “…, especially those residents who require more time or need multiple nurses; they are just left in the bed and not being lifted out of it.”

*Challenges associated with the leadership* refer to a passive and absent leader with poor understanding of nursing. The participants brought up challenges related to coordinating work across different tasks and roles, emerging as lack of planning, unclear job descriptions and the need to perform several tasks simultaneously. Challenges associated with information flow included insufficient documentation of care and communication failures among colleagues. Further, the inadequate induction of new employees was reflected by a lack of clarity to their duties. It was also mentioned that nurses constantly struggle to allocate resources for induction.

P8: “When there aren’t proper inductions, substitutes don’t have the knowledge of what needs to be done. But we can’t keep inducting new nurses all the time.”

The participants described challenges associated with the availability and retention of nurses, which led to difficulties in finding substitutes to cover nurses’ absences. Working with understaffing and frequently changing short-term substitutes was cited as the reasons behind the need for prioritization decision-making. The allocation of limited resources was reflected by the small number of nurses relative to the amount and demands of the work, and insufficient resources for unexpected situations, such as residents’ seizures, was mentioned. The participants reported challenges regarding employee structure as a lack of registered nurses and staff performing non-nursing tasks. Additionally, there were employees providing nursing care without professional education.

### Prioritizing nursing care in decision-making

The basis of the prioritization of care in decision-making among nurses working in nursing homes could be described using three main categories. These were prioritizing the interests of residents, striving for an efficient workflow and prioritizing nurses’ personal interests (Table 2).

**Table 2.** The basis of prioritization decision-making by the participating nurses (*n* = 23).

Subcategory	Upper category	Main category
Empathizing with the resident Understanding residents’ needs	Individualized consideration of the residents’ needs	Prioritizing the interests of residents
Ensuring residents’ well-being and functional capacity Maintaining residents’ health status	Residents’ care plan	
Avoiding harm Avoiding suffering Avoiding jeopardizing residents’ safety	Avoiding adverse outcomes for the residents	
Prioritizing fast, ambulatory residents for nursing care Bedridden residents and those requiring recourses from multiple nurses are treated last	Prioritization of nursing care for low-maintenance residents	Striving for an efficient workflow
Providing care to residents based on room order Rigid routine of nursing care	Repetitive working method that does not require decision-making	
Considering rescheduling Assessment of the impact of the decision on the future workload	Task prioritization in relation to future workload	
Work experience Education	Professional competence	Prioritizing nurses’ personal interests
Intuition Personal hierarchy of importance	Intuitive prioritization	
Avoiding of being caught by colleagues Avoiding consequences from resident complaints	Avoiding negative consequences	

The participants stated that *prioritizing the interest of residents’* manifests as the individualized consideration of the resident’s needs by putting themselves in their position. Nurses were able to perceive the needs of residents they are familiar with, such as thirst or the need to visit the bathroom, even if the resident was unable to express themself.

P9: “I always think that if I were the residents myself, it would be really uncomfortable to sit in a wet diaper.”

Prioritization decision-making was guided by a resident’s care plan, which included methods for ensuring well-being and functional capacity, such as hygiene and nutrition, and maintaining health status. The participants emphasized that their prioritization decision-making was influenced by the aim to prevent adverse outcomes for the resident, including harm, suffering, and jeopardizing their safety.

*The striving for an efficient workflow* was reflected by prioritizing the care of ambulatory residents or those with fewer needs and leaving the more complex residents for last. The participants described a repetitive working method that involved a systematic, room-by-room order approach to resident care to enhance efficiency and reduce transition times. A rigid routine manifested as providing care to residents throughout the day in the same order. The participants stated that they assessed their decisions’ impact on their future workload, emphasizing prioritizing actions that may later lead to an increased workload.

P18: “I try to look at it like, if I know one resident takes more time, I’ll go there last, just so I can get the morning or evening tasks done for as many residents as quickly as possible.”

P1: “I think about what might make me ever busier in a little while. For example, if I don’t change that dirty diaper now, the resident might have soiled themself with stool within an hour, and then I’ll have more work.”

When describing *prioritizing nurses’ personal interests*, the participants stated that their decision-making is rooted in their professional competence, which they developed through education and work experience. It was also influenced by intuition and personal importance hierarchy. The participants related that they avoided negative consequences, such as getting caught by colleagues for misguided prioritizing and unfinishing nursing care, which led them to prioritize visible care activities. In addition, the care of residents who could potentially complain was prioritized.

P15: “I also consider what my colleagues might notice. People tend to judge each other’s work, so by doing visible tasks, you always protect your own back.”

### Support for prioritization decision-making regarding unfinished nursing care

The participants brought up the non-existing support from their work community regarding prioritization decision-making and unfinished nursing care. According to them, the topic was rarely or not at all discussed in their work communities. There were no established operating procedures for them. They reported that, in some work communities, it was openly decided to carry out only the mandatory nursing care activities, for instance, when there was a lack of human resources without informing the leader. Further, the mandatory nursing care activities were defined by the individual nurse and not the community as a whole. It was reported that, typically, residents were left in their beds instead of being assisted to sit up, their dental hygiene was neglected, and social care activities were left undone. The participants stated that prioritization decisions were not openly discussed among their colleagues and that they did not want to make their prioritization decisions visible of public, as they would have become known to others in their work community. Moreover, they did not want to highlight how they made their decisions, as this could attract negative attention or criticism. It was mentioned that new employees quickly learned this culture.

The participants reported a high barrier to bringing up daily unfinished nursing care activities. They did not mention it because they felt embarrassed, ashamed and inadequate. They mentioned activities that they were unable to carry out if these activities were not performed daily and had been pre-planned, such as flushing a urinary catheter. The discussions conducted about unfinished nursing care were mainly at a general level and focused on activities that should receive more attention, such as brushing residents’ teeth. Discussions on the topic in work communities were limited to occasionally blaming colleagues for unfinished activities and making snide remarks about them.

P2: “We don’t openly discuss this. We have some unspoken agreements if there’s a shortage of nurses, such as social care not being carried out and residents will stay in their beds. The leader is definitely not aware of these.”

## Discussion

The aim of the study was to describe prioritization decision-making regarding unfinished nursing care in nursing homes. Previous research on this topic has been limited to examining nurses’ and physicians’ experiences of prioritization^6^ and the factors that influence nurses’ decision-making process.^7,8^ This study not only confirms and supplements the existing knowledge, but also uncovers new insights. It conceptualizes prioritization decision-making regarding unfinished nursing care in nursing homes and makes it visible, thus enabling its development. The findings revealed that prioritization decision-making and unfinished nursing care are rarely discussed in nurses’ work communities, and the issue is covered up. For the first time, the findings identified a negative culture and passive attitude of nurses’, which caused the need for prioritization. This study also found that nurses strive to achieve an efficient workflow by employing a repetitive working method and to safeguard their own interests. The support received by nurses in this context has not been previously investigated, and this study revealed it to be lacking, as the phenomenon is not openly discussed, and common protocols regarding prioritization decision-making have not been established.

### Reasons causing the need for prioritization decision-making

According to the findings of this study, the reasons causing the need for prioritization decision-making were as follows: challenges associated with nurses’ engagement with person-centered care, the culture of the work community, the burden due to workload and challenges associated with the leadership. The reasons linked to the nurse’s competence, attitude, and willingness to develop were a novel finding. The participants seemed to engage in prioritization due to the actions of their colleagues and not their own actions. This raises the question of whether nurses realistically assess their own actions or shield themselves from the ethical burden by perceiving their behavior as being different from their colleagues.

The findings indicated that a nurse’s inability to be present with the residents and their treatment of residents as an objects of nursing care raise concerns about the implementation of person-centered care. The purpose of this approach is to enable the best possible quality of life for individual residents by recognizing and accepting them as they are.^14^ The findings of this study support past research; according to a previous study, residents of nursing homes expressed the need to adjust to the norms of nursing homes and the caregivers’ decision-making.^15^

The reasons associated with nurses’ resistance to change highlight the need for empowering in-work training. Such training should enhance nurses’ professional competences as well as their willingness and capabilities to provide person-centered care to improve the quality of care. Such training might also encourage nurses’ to step out of their comfort zone and enhance their motivation to work.

This study generated new insights into the work community’s culture, identifying it as a reason for the need for prioritization decision-making. The impact of a challenging culture as a potential reason for unfinished nursing care in nursing homes has been previously observed,^16^ but it has not been studied in relation to prioritization decision-making. The participants highlighted several negative attributes of the work community, such as an atmosphere of fear. They emphasized that they did not dare to resist their working community, which limits development and psychological safety. This is understandable, as when people deviate from the norm, they can face criticism.^17^ These factors and the identified idealization of surviving alone are indicative of a lack of trust and support within the work community. According to a systematic review of reviews,^18^ relational leadership has been found to have a positive impact on team collaboration and enhancing nurses’ trust in their colleagues and leader. The leader must enable a work environment where nurses can freely participate, express their opinions and, thereby, enhance the safety and quality of care. This is achievable through transparent management, reward and recognition, effective communication, functional teamwork and ensuring adequate resources.^19^

The participants expressed that the burden due to workload included irregular working hours and challenges associated with recovery. These can be mitigated by planning shifts ergonomically and including recovery breaks from work in advance. A lack of assistance staff to perform non-nursing tasks was identified as a reason causing the need for prioritization decision-making. The participants sated that when they are conducting non-nursing tasks, they do not utilize the professional education they have received. It is paradoxical that amidst a shortage of professional nurses, a part of their expertise is utilized for tasks unrelated to nursing care. Leaders should address role overlaps and ensure that nurses are working within their skill set and education level.^20^

The findings revealed challenges in leader’s actions and skills. In this regard, in addition to unclear job tasks and descriptions, as well as unevenly distributed workloads, inadequate work planning and communication were observed. Leaders could potentially have impact on these reasons. It has been previously stated that relational leadership positively affects, for example, organizational culture and practices, staff collaboration, and the quality of work environment, which includes ethical values and resolution of ethical dilemmas.^18^ Furthermore, the decreased availability of nurses was evident from the findings of this study. Working with short-term substitutes was experienced as burdensome, and insufficient induction of new employees was observed. Leaders should ensure a high-quality induction for substitutes and allocate sufficient resources for the same. This step could potentially alleviate the burden created by delegating responsibilities and tasks to permanent nurses.

### Prioritizing nursing care in decision-making

According to the findings, the prioritization decision-making is based on prioritizing the interests of residents, striving for an efficient workflow and prioritizing nurses’ personal interests. This study demonstrated that prioritizing residents interests included consideration of their needs and care plan and avoiding adverse outcomes. The findings support previous research,^8^ which indicates that nurses in nursing homes prioritize residents’ care needs, the urgency of situations, and anticipation of consequences.

This study generated new insights related to striving for an efficient workflow, as this perspective has not been previously explored within this context. The findings revealed that nurses prioritize care of residents whose care is quicker and easier to perform. Therefore, this raises concerns about the adequacy and quality of nursing care received by those residents who need more time-consuming care. The repetitive working method, which includes proceeding in a room-by-room order, was described, and it does not appear to be person-centered. Shifting the focus from striving for an efficient workflow to enhancing the provision of nursing care that aligns with residents’ needs is essential. The repetitive working method does not require explicit decision-making, as it occurs automatically. It is important to ensure that the prioritization decision-making is visible within the work community and that is discussed collectively. Leaders should involve nurses in the decision-making and dialogue about altering working methods, thus fostering open communication. Through change management strategies, it is possible to transform working methods and culture of the work community. In change management, it has been recognized that factors such as support, communication, measurement, and supervision of change implementation and providing feedback to individuals have a positive impact on the implementation of changes.^21^

The avoidance of negative consequences by the nurses themselves was an entirely new finding. Participants described prioritizing care of residents who could potentially complain. This observation differs from previous studies, which identified that individuals requiring care, such as those with strong opinions or judgmental attitudes, may be more vulnerable to lower prioritization and potentially unfinished care in context of acute care.^22^ This study revealed that prioritization decision-making is also influenced by the need to avoid getting caught by colleagues, which was also a new discovery. Increasing the trust and support among nurses, along with establishing open communication, could potentially guide prioritization to focus more on the residents’ needs rather than the fear of colleagues.

### Received support in relation to prioritization decision-making

The support nurses received for prioritization decision-making and unfinished nursing care was nonexistent. Previous studies have not examined the support received by nurses for prioritization decision-making in nursing homes. However, a previous scoping review highlighted the nurses’ need for ethical support in priority settings^5^ stating that it should be considered at the national level, including nurses’ education and ethical guidelines. A culture that encourages an open dialogue can reduce nurses’ ethical burden related to prioritization.

With regard to the reduction of nurses’ ethical burden, leaders should collaborate with nurses to create a policy for situations requiring prioritization decision-making in nursing homes.^16^ The policy should also consider how prioritization decision-making and the potential unfinished nursing care that it results in are documented. It is essential to monitor and evaluate the functionality of the developed policy. The leaders should also be able to support the nurses and bring up the issue openly during discussions.^23^ Open discussion about prioritization decision-making and unfinished nursing care would serve to improve the quality of nursing care and lighten the ethical burden of nurses.

## Strengths and limitations

The strengths of this study include its wide geographical coverage across Finland, with participants from nursing homes in both the public and private sectors, thus representing diverse working communities and cultures. Therefore, the participants can be regarded as key informants from different care contexts.^24^ Despite the diversities in the working communities, cultures, and employers of the participants, the study’s findings were consistent. Data saturation was achieved during the interviews, after which the data collection was concluded. The data analysis reached a high level of abstraction, enhancing the transferability of the research findings and enabling the examination of the phenomenon in similar contexts.^24^ The data were revisited, and the compatibility of the categories was examined constantly with the original data and research questions to ensure the validity of the analysis.^24^ Discussion about the analysis also took place at each stage with members of the research group.^24^ The inclusion of authentic quotes from the data enhances authenticity and credibility of the study.^25^

However, there are certain limitations to consider. The recruitment process relied on social media, which excluded individuals who did not use these platforms. The research topic is sensitive, a point that was also highlighted by the participants, which might have resulted in their excluding some information during the interview. Further, the findings may have limited transferability beyond the context of Finnish nursing homes, as the employee structure and job descriptions in other countries’ nursing homes may differ. Another limitation of this study is that the chosen analytical method does not allow for a more in-depth interpretation of the data. Hence, in future research on the topic, it is advisable to consider various approaches and methods that could enable a deeper understanding of the research data.^26^ The credibility of the study could also have been strengthened by returning the research findings to the participants.

## Conclusion

This study produced an in-depth understanding about nurses’ prioritization decision-making regarding unfinished nursing care in nursing homes and produced concepts that enable the consideration of the topic. The findings revealed an ethically challenging and hidden phenomenon. Nursing leaders need to be aware of the prioritization decision-making in practice, establish an open policy for it among nurses and make the phenomenon visible to find new ways to support nurses and alleviate their ethical burden. The findings of this study can be utilized for the development of care in nursing homes, for the education of nurses and nursing leaders, and for the development of new approaches to reducing nurses’ ethical burden and for the promotion of person-centered care for nursing home residents. In the future, it is vital to investigate the ethics involved in prioritization and the effect of organizational culture on nurses’ decision-making.
